# Virtual Enactment Effect on Memory in Young and Aged Populations: A Systematic Review

**DOI:** 10.3390/jcm8050620

**Published:** 2019-05-07

**Authors:** Cosimo Tuena, Silvia Serino, Léo Dutriaux, Giuseppe Riva, Pascale Piolino

**Affiliations:** 1 Applied Technology for Neuro-Psychology Lab, IRCCS Istituto Auxologico Italiano, 20149 Milan, Italy; 2 MySpace Lab, Department of Clinical Neurosciences, University Hospital Lausanne (CHUV), CH-1011 Lausanne, Switzerland; silvia.serino@chuv.ch; 3 Institute of Neuroscience and Psychology, University of Glasgow, Glasgow G12 8QB, UK; leo.dutriaux@glasgow.ac.uk; 4 Department of Psychology, Catholic University of the Sacred Heart, 20123 Milan, Italy; giuseppe.riva@unicatt.it; 5 Memory and Cognition Laboratory, Institute of Psychology, Paris Descartes University, Sorbonne Paris Cité, 92774 Boulogne-Billancourt, France; pascale.piolino@parisdescartes.fr; 6 INSERM UMR S894, Center for Psychiatry and Neurosciences, 75014 Paris, France; 7 Institut Universitaire de France (IUF), 75231 Paris, France

**Keywords:** spatial memory, episodic memory, virtual reality, enactment, memory rehabilitation, embodied cognition, aging

## Abstract

Background: Spatial cognition is a critical aspect of episodic memory, as it provides the scaffold for events and enables successful retrieval. Virtual enactment (sensorimotor and cognitive interaction) by means of input devices within virtual environments provides an excellent opportunity to enhance encoding and to support memory retrieval with useful traces in the brain compared to passive observation. Methods: We conducted a systematic review with Preferred Reporting Items for Systematic Reviews and Meta-Analysis (PRISMA) guidelines concerning the virtual enactment effect on spatial and episodic memory in young and aged populations. We aim at giving guidelines for virtual enactment studies, especially in the context of aging, where spatial and episodic memory decline. Results: Our findings reveal a positive effect on spatial and episodic memory in the young population and promising outcomes in aging. Several cognitive factors (e.g., executive function, decision-making, and visual components) mediate memory performances. Findings should be taken into account for future interventions in aging. Conclusions: The present review sheds light on the key role of the sensorimotor and cognitive systems for memory rehabilitation by means of a more ecological tool such as virtual reality and stresses the importance of the body for cognition, endorsing the view of an embodied mind.

## 1. Introduction

When we think of an event, we commonly see with our mind’s eye where this event occurred and what temporal, perceptual, and affective details were associated with it; indeed, this spatial scaffold influences the specificity, richness, and vividness of events we retrieve from the memory [1]. When not defined in its schematic representation of the topography, this ability is considered as the ability to visualize the detailed spatial context (e.g., street, room, park) of specific episodes [2]. In its topographical definition, spatial memory [3] is a complex ability devoted to the encoding and storage of different types of information from our surroundings for successful orientation and navigation. Spatial information is represented and used in our brain with two frames of reference [4]: egocentric (self-to-object) and allocentric (object-to-object), respectively located in the parietal and medial temporal regions with the retrosplenial cortex, playing a critical role in switching between these representations [5]. Spatial information can be divided into survey (e.g., maps, wayfinding, and pointing task), route (e.g., dynamic sequencing of landmarks), and landmark knowledge (e.g., landmark recognition) [6]. Survey knowledge refers to an allocentric map of the spatial layout, whereas route and landmark knowledge are based on an egocentric representation of the space. 

On the other hand, episodic memory is a neurocognitive system that allows people to remember the *what*, *where*, and *when* of a personally experienced event [7]. Binding [8,9,10] is a key feature of this system; it is the process that binds the *what* with the other contextual features (i.e., *when*, *where*, and *details* such as perceptual and affective details). These elements are crucial for the so-called “autonoetic consciousness”, or the feeling of mentally travelling back to the spatiotemporal and phenomenal features of the experienced event [7,11,12].

The hippocampus is known to play a crucial role in spatial cognition [4,13,14], episodic memory [15,16], and recognition [17]; this structure binds cognitive, bodily and emotional information [18,19,20] and connects to cortical representations facilitating the retrieval of episodes [21]. In particular, according to Nadel and colleagues [13,22] the hippocampus provides the allocentric spatial scaffold for episodes binding neocortial representations of the event (i.e., Multiple Trace Theory). The link between spatial cognition and episodic memory is also highlighted by the fact that egocentric spatial updating with self-motion cues (i.e., path integration of dynamic bodily signals) plays a critical role during retrieval (recall and recognition) of dynamically encoded scenes [23], confirming the role of egocentric information in manipulating and translating allocentric long-term representations of events [24,25]. Despite the crucial role of medial temporal lobes during encoding, storage, and retrieval [26], the parietal and frontal lobes have been also identified as a crucial substrate of episodic memory, absolving different declarative memory functions such as encoding, retrieval, storage, and monitoring [27,28,29,30] (for a meta-analysis of navigation and episodic memory brain network, see [31]). 

Recent insights from philosophy, psychology, and neuroscience have drawn attention to the essential role of the body in cognition [32,33]. The framework known as the “embodied cognition” theory provided a fresh and innovative way to conceptualize the relationship between these two long-debated components of human psychology. Indeed, psychological processes are influenced by body morphology and sensorimotor systems [34]. There is growing interest and evidence on how the body affects several cognitive domains, including memory [35,36]. However, the concept of memory can be expanded to take into account the whole body as crucial in encoding, storage, and retrieval [37]. These assumptions have great relevance in the context of normal and pathological aging, where physiological changes modify regions of the brain involved in memory formation, leaving primary cortices spared [38,39,40]. 

Indeed, sensorimotor involvement may leave traces that are useful for memory retrieval [41,42,43], and encoding strategies are among the most effective methods to enhance memory [8]. The encoding specificity principle states that recollection is facilitated when an overlap occurs between the elements of the retrieval context and those of the encoding context [44]. Retrieval is possible thanks to a cue, and a memory trace is mediated by the same cognitive operations that occurred during encoding [45]. From a neuroanatomical point of view, there is growing theoretical and empirical evidence indicating how retrieval may be considered an overlapping process [46,47] that reactivates the same brain regions at encoding [21,48,49], including primary cortices [50,51,52].

Interestingly, active navigation in virtual environments (VEs) by means of input tools can be considered a form of enactment able to enhance spatial [41] and episodic [8] performance. According to Wilson and colleagues [53], active navigation in VEs can be divided into physical activity (motor control) and psychological activity (decision-making). More precisely, the manipulation of spatial information is not the only process involved in navigation; rather, motor commands, proprioceptive information, vestibular information, decision-making, and allocation of attentional resources are all also essential parts of what is called “active spatial learning” in everyday life, whereas passive navigation involves visual information only [6]. We define the virtual enactment effect as the effect provided by one or more of these components on memory retrieval compared to the virtual passive observation of the environment. Virtual reality (VR) allows individuals to interact with the environment thanks to multimodal stimulation, providing a rich embodied experience [54] that can be used to enhance memory in elders [55]. Indeed, technological devices (e.g., joysticks or 3D visors) require the subject to process psychological information, as well as idiothetic (i.e., motor commands, proprioception, and vestibular information) and allothetic information (e.g., landmarks and boundaries). The aim of this work is to review the potential of the virtual enactment effect (i.e., the role of active components of virtual navigation compared to passive observation) in order to contribute to a better understanding of its beneficial effect on spatial and episodic memory. This contribution will provide research and clinical guidelines for future studies within the context of VR memory rehabilitation and enhancement. In order to provide a complete overview of the results, we will cluster findings according to spatial memory (survey and route and landmark knowledge; respectively allocentric and egocentric frames) and episodic memory tasks (episodic features, such as *what*, *where*, *when, details*, and binding; episodic functioning, like learning, forgetting, and strategic processing; and item recognition). 

## 2. Method

Preferred Reporting Items for Systematic Reviews and Meta-Analysis (PRISMA) guidelines were followed [56].

### 2.1. Search Strategy

**Figure 1:**
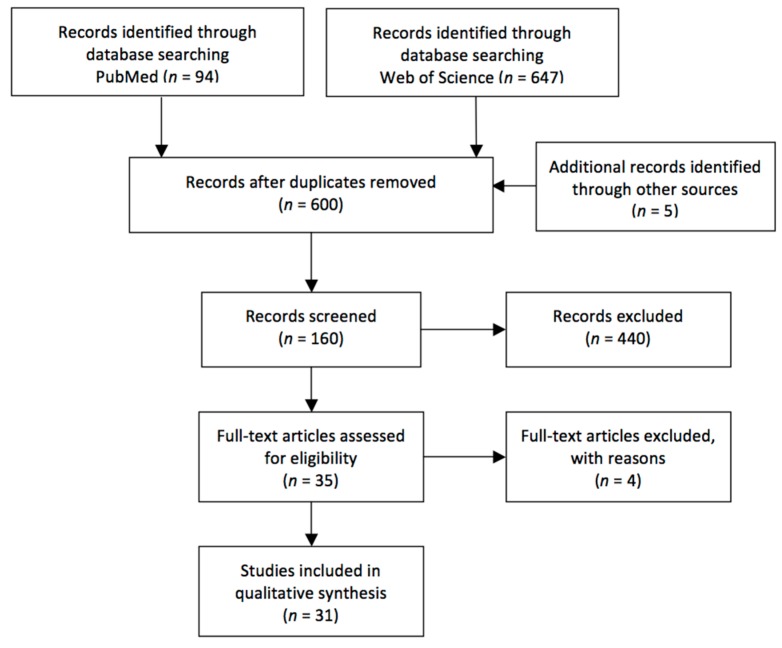
PRISMA flow chart.

Two high-profile databases (PubMed and Web of Science) were used to perform the computer-based research on the 25 January 2019. The string used to carry out the search (Title/Abstract for PubMed and Topic for Web of Science) was as follows: (“active” OR “enactment”) AND (“spatial memory” OR “spatial knowledge” OR “episodic memory”) AND (“virtual reality” OR “environment*). The search resulted in 647 articles for Web of Science and 94 for PubMed (total of 741). We made a first selection by reading titles and abstracts after removing duplicates. Four papers were identified through other sources. A total of 35 manuscripts were chosen for full-text screening. This procedure resulted in 31 experimental studies. See the flow diagram (Figure 1) for the paper selection procedure.

### 2.2. Selection Criteria

Studies on the role of active navigation and enactment on spatial and episodic memory in young and aged populations (healthy and pathological) were included. We also included studies in languages other than English and excluded studies which did not follow our aims (non-age-related diseases, developmental studies, active- or passive-only conditions, active vs. passive conditions not related to the context of active navigation and action). We excluded articles for which the full text was not available or for which the abstract lacked basic information for review. Reviews, meeting abstracts, notes, case reports, letters to the editor, research protocols, patents, editorials and other editorial materials were also excluded. Five studies [57,58,59,60,61] did not appear during our search but were in line with our inclusion criteria; therefore, they were added to the included studies.

### 2.3. Quality Assessment and Data Abstraction

PRISMA guidelines were strictly followed; search results found by the first author (C.T.) were shared with the review authors for individual selection of papers in order to reduce the risk of bias, and disagreements were resolved through consensus. The data extracted from each included study were as follows: reference, year, sample(s), conditions, design (for the navigation condition), virtual apparatus, memory assessment, and primary outcomes.

## 3. Results

Several studies have been conducted to assess the role of active navigation in human memory. However, the growing interest in virtual reality (VR) has led researchers to question how the different aspects of navigation interact with the virtual environment. In particular, sensorimotor involvement, which is known for its positive effect on memory enhancement, seems to be one of the most investigated virtual enactment form. In our review, we aim at discovering whether this beneficial effect could also be observed when the subjects interact with technology devices.

To satisfy our aim, six clusters will be discussed: (1) the target population; (2) virtual apparatus; (3) conditions manipulated during navigation; (4) memory tasks; (5) the role of action and its effects on memory; and (6) cognitive domains underlying active navigation and memory performances. A synthesis of the results is reported in Table 1. Nine studies in Table 1 are reported with each sub-experiment; among these, only the experiments (e.g., Exp. 1, Exp. 2) that aim specifically at studying spatial or episodic memory appear in the table.

### 3.1. What Populations Have Been Included?

From our systematic search, it emerged that the majority of the experiments included healthy participants, mainly young adults (YA), but also older adults (OA). Studies focused on spatial domain; however, a cluster of seven studies investigated episodic memory and its subcomponents in healthy populations (YA and OA). Nevertheless, age ranges varied across the studies for YA and OA, and for the “student” samples the age information was vague. Importantly, in six studies no gender information [53,63,68,71] or matching [58,82] were reported. Only one study recruited clinical populations of Alzheimer’s disease (AD) pathology: 15 AD patients and 15 amnestic mild cognitive impairment (aMCI) patients compared to 21 healthy OA were included in the study of Plancher and colleagues [70] to assess the effect of active and passive virtual navigation on episodic performance. A synthesis of populations (YA, OA, AD, and aMCI), with mean age and standard deviation and number of males/females, is reported in Table 1.

### 3.2. What Virtual Apparatus Have Been Used?

For the purposes of our review, it is essential to summarize the apparatus been used in each experiment. Ecological virtual environments (VEs) have been used to assess the virtual enactment effect regardless of the domain (spatial or episodic memory); specifically, cities or apartments were used to evaluate the effect of active interaction (e.g., input device interaction)—namely, the “virtual enactment effect”—on memory recall, while four experiments [9,53,67,74] used basic virtual scenarios with poor ecological validity (e.g., virtual arenas). Concerning the input devices, researchers mainly used joysticks and keyboards to navigate the VEs, whereas five studies used a steering wheel and pedals to control a virtual car. The use of these controllers is linked to the type of immersion; indeed, the vast majority of the experiments’ apparatus were non-immersive (PC screen or projectors). Only six experiments [57,58,61,73,79,80] used head-mounted display (HMD) to assess the role of active navigation on spatial performances and only one study used immersive virtual reality to assess the effect of full body involvement during encoding on episodic retrieval.

### 3.3. What are the Navigation Conditions in the Included Studies?

In the following paragraph, studies will be discussed in terms of navigation condition, degree of decision-making, and type of encoding. Active navigation studies used classic dynamic navigation, whereas non-dynamic navigation (e.g., snapshots or teleporting) were added as the comparison condition [62,67,80]. The former might be more suitable compared to static navigation if we consider the role of constant mapping provided by the hippocampus (i.e., place cells) in building the map of the environment [83]. Passive navigation in the studies included in the review consisted of a yoked condition or pre-recorded navigations. Navigational decision-making, or free exploration, is another crucial aspect of active navigation and spatial knowledge [84]; however, in 17 experiments [9,59,60,61,62,63,64,65,70,71,72,77,78,79], researchers gave a predetermined route or instructions to follow. Moreover, decision-making is a crucial aspect of the virtual enactment effect when older participants are involved in active navigation [8] due to overload on the frontal lobes and the executive functions capacity on memory encoding [68], which are known to decline with aging [85]. Indeed, Jebara and colleagues [8] found that navigational decision-making, intended as a form of virtual enactment effect, is more effective in OA compared to the active motor condition due to executive function overload at encoding [8,63,68]. Another aspect to consider is the point of view (areal vs. egocentric). The egocentric point of view along combined with the active motor condition improves allocentric and egocentric memory, whereas the areal point of view with passive navigation improves allocentric memory only [60]. Graphic realism when building VEs should take into account the fact that a detailed environment positively affects memory performances [77]. Other elements that neuroscientists in the field of VR and memory should consider are the type of encoding (incidental vs. intentional); although the authors of [62] showed no effect of encoding, in our review intentional encoding leads to better performance across the populations and the type of memory assessed [8,9,43,58,59,67,68,69,70,75,80]. Crucially, only one study [72] compared active VR vs. passive VR vs. real-world navigation, with real-world navigation and active VR leading to better spatial recall, in this order, compared to passive VR. Lastly, from a methodological point of view, researchers are encouraged to evaluate the consequences of using between or within condition. In our review, the majority of the studies included between navigation conditions, while only five studies [57,61,62,67,79] used within conditions. Researchers should consider strengths and weaknesses of within and between designs with potential biases in the light of the objectives of their study [86], as explained in the discussion paragraph. 

### 3.4. How Has Memory Performance Been Measured?

Several VEs (cities, rooms, mazes, and arenas) were used in the reviewed experiments in order to test two main memory clusters: spatial memory and episodic memory (event and object memory). The evaluation included for spatial memory tasks involved survey knowledge (maps, pointing and wayfinding tasks), route knowledge (chronological order tasks), and item recognition/recall for landmark knowledge (see Table 1 for a summary of these tasks).

To investigate the role of active navigation in episodic memory, six studies used a similar navigation paradigm in a virtual city in which events occurred [10,43,57,70,71]. Participants were tested on events encountered, landmarks and spatial layout of the cities. These paradigms aim at assessing *what*, *where* (egocentric and allocentric), *when*, *details*, and binding among elements in ecological VEs with free recall, delayed recall, and recognition. Laurent et al. [9] studied the different components of episodic memory; a variation of the WWW (*what*–*when*–*where*) task was used in order to study the binding of contextual aspects to objects. Finally, Sauzéon and colleagues [68,69] used free recall (learning, proactive interference, semantic clustering based on the California Verbal Learning Test (CVLT) [87]) and a recognition task (recognition hits and false recognitions); object recognition memory was also used to assess event memory in Pettijohn and Radvansky [82]. Interestingly, Pacheco and Verschure [81] assessed their samples on an immediate and delayed free recall task of images semantically associated with an object they found in the virtual town. For a summary of these tasks, see Table 1.

### 3.5. Do “Virtual” Actions Have a “Real” Effect on Spatial and Episodic Memory?

**Figure 2:**
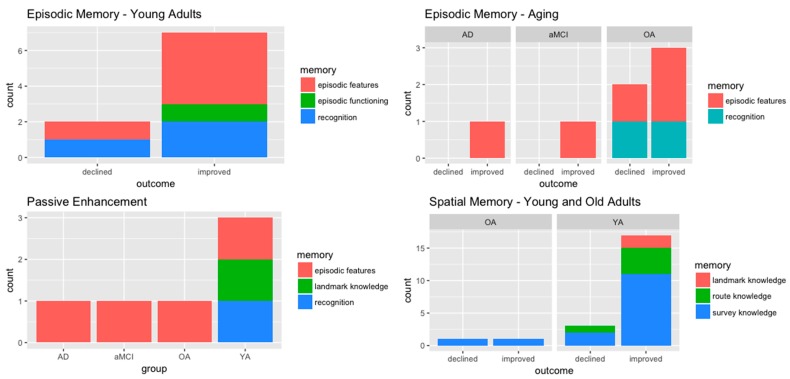
Summary of virtual enactment effect and passive enhancement in the samples. YA: young adults; OA: older adults; aMCI: amnestic mild cognitive impairment; AD: Alzheimer’s disease.

In the following subsection, memory performance will be clustered by the different components taken into consideration in the included study of the review: spatial memory, episodic memory, and recognition memory of both spatial and episodic studies (Figure 2). The Primary Outcomes column in Table 1 provides in detail the virtual enactment effect on each task/measure. Using the correct task to target specific sub-components is crucial for the researchers; we suggest future research to put effort into designing and conceptualizing the task and test method to tap memory processes. In the present review, we found a general positive effect of virtual enactment in young adults for spatial memory; however, further studies need to assess this in older adults as spatial enhancement in OA is controversial [61,63]. In particular for spatial scores in aging, active navigation involving an overloading task during encoding affected retrieval [63]; decision-making in active navigation appeared to be more suitable in this sample [8].

Similarly, experiments investigating episodic memory showed initial support for a virtual enactment effect in young adults (Figure 2); although findings are few, encouraging results come from studies of neurodegenerative conditions that may benefit from virtual enactment, whereas non-spatial features of episodic memory are influenced by demanding tasks during encoding (Figure 2). These findings could also confirm the embodied nature of episodic memory as a cognitive and bodily experience [18,20,35,88,89,90].

Initial recognition scores in spatial and memory performance results are controversial and need further investigation. Although past research on enactment showed a positive effect on recognition memory [91], recognition scores in spatial and memory performance results are controversial. A possible explanation for this could be that recognition occurs in the brain at different degrees [92,93] such as visual recognition, guessing (Guess responses), familiarity (Know responses), and source recognition and recollection (Remember responses); the latter, with source memory, is thought to be related to recollective aspects of episodic memory linked with autonoesis and full detailed recall. As a consequence, it is important to adopt a recognition paradigm that is able to grasp perceptual and sensory elements of the memory traces at retrieval.

No effect was found in these spatial studies [53,66,74,78,79], and on episodic memory scores (*what*, verbal *where*, visuospatial *where*, *when*, and *details* in the works of Plancher et al. [71] and Tuena and colleagues [57]), or on object recognition memory in the work of Pacheco and Verschure [81]. Finally, although results are encouraging, some studies showed passive enhancement (see Figure 2); therefore, results of the review are preliminary, and future studies need to deepen the virtual enactment effect in order to confirm the enhancing effect from which different populations might benefit.

It is well known that active navigation promotes better learning performance [6]. We found confirmations of how the body shapes memories and how it can be used as a medium to enhance learning by means of input tools as an extension of previous research on the enactment effect with ecological scenarios and items [42,88,94,95].

### 3.6. What Are the Cognitive Factors Mediating Active Navigation and Memory Performance?

Among the included studies, cognitive factors underlying navigation have been studied in the same healthy population [74], whereas neuropsychological factors were used to evaluate the effect of active navigation on memory in YA vs. OA [8,63]. In particular, visuospatial abilities and especially executive function seem to be crucial for spatial memory [63,74] and for episodic memory functioning [68] and features [70,71]; in particular, executive function and attention appear to influence performance [8,69].

Cutmore et al. [74] conducted four experiments in order to evaluate the effect of gender, visuospatial abilities, cognitive style, and cerebral asymmetry during static active navigation (teleporting). Males showed faster results in finding the exit of the virtual maze compared with females, while both groups benefited from a landmark cue condition (landmark associated with a room). Moreover, males were more accurate; in this case, a compass cue condition (compass heading cue) led to better performance compared to a landmark condition. Visuospatial abilities were evaluated with the Wechsler Adult Intelligence Scale—Revised (WAIS-R) [96]. The high visuospatial group was better at Euclidean (survey knowledge) distance estimation compared with the low group. Participants’ cognitive styles (verbal-sequential vs. visuospatial) were evaluated with the same test. The visuospatial group showed better navigation performance; moreover, this effect was shown for static navigation when compared with verbal-sequential participants. The visuospatial group was also better in navigating the maze backward. Finally, Cutemore and colleagues [74] used electroencephalography to observe cerebral asymmetry: the verbal-sequential group showed a greater right hemisphere activation (effort computing spatial problem solving) compared with the visuospatial group. However, only females were recruited for the last three experiments, since a gender effect was found in the second experiment. The authors wanted to evaluate whether navigation is related to superior spatial skills in a sample of females, but von Stülpnagel [88] found that sense of orientation abilities affected false alarms and route navigation performance regardless of gender.

Taillade et al. [63] found that YA were better than OA in terms of executive function, visuospatial abilities, and memory. In particular, wayfinding tasks (survey knowledge) seemed to be affected by executive function. Jebara et al. [8] correlated the binding scores with age and neuropsychological tests. An effect of age was found in all the navigation conditions. Binding score was significantly correlated with visual memory and working memory in high navigation control (HNC; motor trace and decision-making), low navigation control (LNC; motor trace only), and itinerary condition (IC; decision-making only) conditions. Shifting (executive function) negatively affected the VR binding scores for LNC and HNC but not for the passive condition and IC. When controlling for age, the scores in HNC were still significantly affected by executive function. Verbal memory correlated positively only with the IC condition. Similarly, Sauzéon and colleagues found a positive correlation between recognition hits and episodic memory (CVLT [87]) and executive function (mental rotation and Stroop color-word task; [97,98]) in the active but not the passive navigation condition. Total false recognitions (source-based and gist-based) were correlated with episodic memory and executive function tests. An age effect on false recognition was also found. In particular, executive function, after partial correlation between age and false recognitions, contributed to recognition performance under the active navigation condition [68].

It is also worth reporting that cognitive differences among the populations emerged in terms of gender, age and pathology. Plancher and colleagues [70] found differences among individuals with AD, aMCI, and OA in their episodic memory task. The same performance pattern (AD < aMCI < OA) emerged for *what*, *details*, egocentric and allocentric *where*, and recognition. AD and aMCI individuals had less recollection compared to controls. AD patients’ binding was lower than that of aMCI patients and OA, whereas aMCI patients had a delayed recall deficit compared to OA. *When* scores were lower for the AD group compared with the aMCI group and OA, while AD and aMCI individuals presented a difference between immediate and delayed recall. Moreover, the authors found better *what* recognition in OA, aMCI, and AD compared to other episodic recognitions. Plancher and colleagues found that AD and aMCI patients had worse recognition than OA; similarly, OA performed better in terms of recollection rates than aMCI and AD. 

Tasks revealed an age effect in different studies [8,61,63,71,80] as well as a gender effect [43,65,80]. The findings of Jebara and co-authors [8] revealed an age effect for *what* (immediate and delayed), binding (immediate and delayed), visuospatial recall, and recognition total score. An age effect on spatial memory and recognition was found by other studies. Sauzéon and co-authors [68] showed that OA have worse learning, proactive interference, and false recognition compared with YA. Taillade and colleagues [63] found that YA fared better in a wayfinding task than OA, but this pattern did not emerge for spatial memory tasks. An age effect emerged in the first study of Plancher and colleagues [71]. Young participants were better compared to OA in terms of verbal and visuospatial *where*, *when*, and *details*. Moreover, findings showed a main effect of intentional encoding for *what*, verbal *where*, visuospatial *where*, *when*, and *details*. In an active navigation condition, OA had better *what* recall in incidental encoding, while YA had better recognition, *when*, verbal *where*, and visuospatial *where* on intentional encoding compared with OA. Finally, Plancher and co-authors [56] found that women performed better (statistical tendency) on the recognition task, whereas men had better scores for the cued visuospatial task. No effect of condition emerged. Von Stülpnagel and Steffens [65] showed that women had more false alarms than men, who were faster in a route navigation task; moreover, women revealed a lower sense of orientation and computer experience than men. Findings of Dalgarno et al. [64] were not affected by gender. Lastly, recall decreased with age [68] for both immediate and delayed recall [6]. Taillade et al. [63] highlighted an age effect for a wayfinding task but not for a spatial memory task. Recognitions decreased with age [8], but the same was not found by Sauzéon and co-authors [68], who showed more false recognition for OA. 

The following neuropsychological tests were correlated with memory tasks [71]. Trail Making Test (TMT) A and B [99] was used to evaluate executive functions and attention in OA and was negatively correlated with *what* and *where* and sustained attention scores were associated with *where* responses; lastly, the Cognitive Difficulties Scale [70,71] was significantly associated with episodic scores in normal and pathological aging.

Von Stülpnagel and Steffens [65] found different interactions with the movement (self-contained vs. observed) condition. In the second experiment, providing layout and landmark information along with self-contained movement led to better route knowledge (tour integration task), whereas for the route navigation task better scores were obtained when self-contained movements were associated with reading instructions. In the last of their experiments, recognition performance was enhanced when any allocentric map was given, whereas the tour integration task benefited by a map with path to follow and not by self-contained condition. Finally, in the route navigation, the map with a path to follow worsened the scores, whereas the active map helped participants in the observed movement condition. Farrell and colleagues [76], in their first experiment, found that active navigation with or without an allocentric map led to better virtual to real world transfer of spatial knowledge compared to control (real-world wayfinding) and this is true also for active virtual exploration with a path to follow (no decision-making). In their second experiment, virtual exploration with the map did not lead to better transfer compared to the allocentric map studying condition without real or virtual exploration.

Other relevant effects that interact with cognition are reported. In particular, dynamic active navigation generally led to better results, as noted by three studies [62,67,80]; however contradictory (path shape but not orientation and recognition; [62]) were also reported. Some authors [62,64,80] have highlighted the importance of optic flow for spatial learning in VE. Visual fidelity is also crucial for both survey and route knowledge [77], and the first-person perspective of the virtual environment boosted wayfinding and route knowledge, whereas an aerial-view improved allocentric representation [60]. Three-dimensional virtual reality seems to stimulate memory due to higher body involvement but also reduced energy consumption [74]; however, Palermo and co-authors [79] found that immersive interaction with a gyroscope, although reported as interesting, could be frustrating and showed a minor degree of engagement compared to classic active interaction with a mouse. Finally, a trial effect emerged confirming the positive effect of repetition on performance (e.g., [59]). Interestingly, exposure times led to better survey representation for passive participants, as noted by Sandamas and Foreman [59]. Wallet et al. [72] found better delayed recall, but not Pacheco and Verschure [81], in their 24-h delayed recall, for active participants after 48 h for spatial memory. Similarly, Jebara et al. [6] found YA, but not OA, had better delayed (20-min) as compared to immediate recall for episodic recall (item information and binding). No effect of condition emerged in the latter study. With regard to expertise, Sandamas and Foreman [75] found that drivers, regardless of the navigation condition, were better in the map task (survey knowledge). Moreover, different studies aimed at balancing driving [6,43,75] and technology experience (e.g., [60,65]), since these could influence the performance.

In addition to age, gender, skills, and cognitive functioning, it is crucial for neuropsychology research to consider the roles of consolidation, repetition, and dynamic changes in order to build effective and ergonomic training for memory rehabilitation and enhancement.

## 4. Discussion

In the present review, we provided initial positive results concerning the virtual enactment effect on spatial and episodic memory performance, highlighting the embodied potential of virtual reality (VR). For each of the questions presented (see Section 3 subsection headings), we provided theoretical and practical solutions to guide future studies within the context of the virtual enactment effect and its use in aging. To summarize, the virtual enactment effect on memory is: (1) present in the young population; (2) possible in aging but needs further investigation; (3) mediated by neurocognitive factors, especially in aging; and (4) dependent on the use of technological devices and their interaction characteristics.

In general, we suggest that future research should aim at designing experiments for older people and pathological aging, in both spatial and episodic memory in order to test the virtual enactment effect. Moreover, we encourage further research on episodic memory involving young participants to consolidate or extend the findings we reported in this systematic review. Innovative cognitive rehabilitative systems are needed to slow down or prevent memory decline in neurodegenerative conditions, and VR provides a powerful tool to stimulate brain plasticity in Alzheimer’s disease and aging [100,101].

We highly recommend that researchers take into account these elements and consider the use of immersive apparatus by means of head-mounted display (HMD). The main limits of non-immersive studies reported in the review are that they do not grasp the full experience of active navigation, since they do not involve bodily-based (e.g., idiothetic) components [6], and the motor traces used while using controllers might be too weak to have an impact on memory traces [71]. Therefore, researchers should consider the use of a HMD. For instance, with an HMD it is possible to walk around a small area with trackers detecting movements and interact with the scenario with controllers. Moreover, VR enables the user to experience an “egocentric space” [102], which is a critical aspect of spatial processing as it occurs in everyday life [4]. Researchers should not forget the role of interaction (e.g., intentions and actions) on the sense of presence, which is considered to impact more on presence rather than graphic realism [103]. Another critical aspect of memory performance is the type of encoding of virtual scenarios. In real-life situations, episodic memory encoding occurs non-intentionally [10], whereas with spatial learning a certain amount of information is encoded incidentally and with procedural memory [104,105]. However, when planning interventions that exploit the virtual enactment effect, clinicians and neuroscientists are encouraged to design instructions according to the sample; for instance, aging is known to affect incidental rather than intentional encoding, with attentional and executive components playing a critical role in encoding and storage in the former [106]. Lastly, from a methodological point of view, we encourage the use of within-interaction conditions: first, within-subject studies have greater statistical power compared to between-subjects designs; second, they allow the researcher to control variables (e.g., gender) that may affect memory performance (e.g., gender effect on spatial memory [80]), thus providing balanced groups; finally, within-subject designs permit the researcher to assess source memory by asking the participants to recall the context in which an event occurred (see [57]). However, within-subjects studies might overload or confound memory traces if the tasks are too complex or numerous. Researchers should consider the strengths and weaknesses of within and between subjects designs with potential biases in light of the objectives of their studies [86].

Assessment of performance is a critical aspect of research and clinical practice in order to evaluate and analyse what the researcher really wants to achieve. We highly recommend defining tasks based on strong theoretical and empirical considerations when assessing the complexity of memory within the context of virtual enactment. In the context of spatial memory, we suggest using egocentric and allocentric measures to tap spatial cognition features [4]; this can be achieved using landmarks, boundaries and maps with paper-and-pencil, computerized or VR tasks. However, in the present review, this is especially true when the spatial layout is considered within the context of episodic memory (e.g., [8,57]). When research focused on schematic/topographical representation [2], papers mainly used spatial levels of knowledge of the space (survey, route and landmark); therefore, we applied these levels to the cluster spatial task (Figure 2). Nevertheless, recent discoveries in cognitive neuroscience and clinical neuropsychology support the crucial role of spatial frames of reference in representing the space [5,14,83]; moreover, survey knowledge and landmark knowledge resemble, respectively, allocentric and egocentric representation, whereas route knowledge appears to be related to procedural memory due to landmark-based navigation [105,107]. For episodic memory, we strongly encourage the paradigms that tap the elements described by Tulving [7] as central aspects of this type of memory (i.e., event, spatiotemporal details, and emotional and perceptual details). The advent of VR enables neuroscientists to study in an ecological, standardized, and realistic way a complex function such as episodic memory [8,108].

Concerning the virtual enactment effect on spatial memory, the young population reported more positive outcomes on survey knowledge compared to route and landmark knowledge; nevertheless, findings in general are promising. Young adults and both the healthy and pathological aged population showed improvements on episodic item memory, spatial context and binding. However, further studies need to evaluate this effect on aging and neurodegenerative disorders in the domains of both spatial and episodic memory. It might be of interest to deepen our understanding of in which situations passive enhancement is present and why (Figure 2). Moreover, we suggest that future studies include real-world navigation conditions; while all of the studies had a passive control condition, only one [72] used a real-world control condition. Finally concerning mediating factors, although visuospatial abilities are crucial for spatial memory [74], executive functions have a great impact on spatial and episodic performances, and this is especially true for older people [8,63,68]. Other variables such as age, gender, expertise (e.g., videogames, driving), dynamic navigation, virtual realism, and delayed testing influence memory performance. 

Findings are promising in the light of memory decline in aging. An age-dependent decrease is normally observed in these crucial cognitive domains [109], and the decline in spatial and episodic memory is accompanied by neural changes in the medial temporal lobe, hippocampus and prefrontal cortex in the aged population [110,111,112]. Aging is accompanied by spatial memory decline [113]. Indeed, Colombo and colleagues [114] recently shown that older people have specific allocentric impairments and difficulty in switching between the egocentric and the allocentric frame of reference; the translation from the allocentric to the egocentric frame of reference is possible thanks to the activity of the retrosplenial cortex, which converts neural representations of the medial temporal lobe to parietal and vice versa [5,115].

In particular, spatiotemporal details, along with associative (i.e., medial temporal binding processes) and strategic (i.e., frontal monitoring during encoding and retrieval) information, decline with aging [8]. Aging is also accompanied by differences in the encoding and retrieval of episodic memories [10,11]. Piolino and colleagues [11] showed that this was particularly true for autobiographical events in recent periods, with more responses (less spatiotemporal information, details, familiarity and third-person perspective) associated with reduced autonoesis for older adults (OA) compared to young adults (YA).

Memory impairments due to medial temporal lobe degeneration are classic features of Alzheimer’s disease (AD) [116], mild cognitive impairment (MCI) [117], and amnestic mild cognitive impairment (aMCI) [118], which are considered part of the prodromal stage of dementia and in particular AD [119]. Deactivation and decreased functional connectivity of the default mode network is shown in healthy aging, MCI, and AD [120,121,122]. Retrosplenial cortex hypoactivity occurs in both AD and MCI and may explain episodic and navigation deficits in these patients [5]. Spatial disorientation in AD and aMCI is thought to be the result of degenerative processes taking place in the hippocampus and in deficient spatial frame synchronization [123]. Indeed, early markers of AD can be the switching abilities in aMCI and AD individuals [124]: allocentric impairments are present in aMCI and AD patients and moreover a deficit in the switch from egocentric to allocentric was found in these groups. Concerning episodic memory, these neurological conditions lead to deficits in the spatiotemporal and binding components of episodic recall [70], as well as autonoetic consciousness [125,126]. 

Finally, the present findings stress the essential role of the body in cognition, and memory in particular, as claimed by embodied cognition researchers. The virtual enactment effect could be used to study how the different levels of active and passive virtual navigation contribute to spatial and episodic performance and could potentially be used as a way to enhance memory in aging. 

## Figures and Tables

**Table 1:** Summary of the included studies. VE: virtual environment; HMD: head-mounted display; VR: virtual reality; OA: older adults; YA: young adults; aMCI: amnestic mild cognitive impairment; AD: Alzheimer’s disease; HNC: high navigation control; LNC: low navigation control; IC: itinerary control; Exp.: experiment; //: same as above; 1A: first trial apartment; 2A: second trial apartment; B: third trial apartment.

Ref.	Sample (s)	Conditions	Design (Navigation)	Virtual Apparatus	Memory Assessment	Primary Outcomes
[62]	48 YA (age range: 21–38; 24 males)	Effect of active (no decisional level) vs. passive (prerecorded travel) vs. snapshot exploration (static condition) on scene recognition and memory of displacements. Intentional encoding.	Within	Non-immersive (CaTS driving simulator); input device: joystick.	Scene recognition (route snapshots); Pointing toward the origin test using the joystick; drawing test (shape of the path).	Path shape task benefitted from active condition, whereas recognition and pointing task were not affected by the exploring conditions.
[41] Exp. 1	30 YA (mean age = 27.1; 14 males)	Effect of active vs. passive (recorded navigation) navigation on spatial memory. No intentional encoding. Participants could freely navigate the apartment.	Between (yoked)	Non-immersive; input device: joystick; house apartment navigation.	Spatial layout test (spatial layout drawing of the VE); recall test (location and objects name on VE map).	Active group showed better spatial layout scores. No effect on recall test.
[41] Exp. 2	40 YA (mean age = 26; 18 males)	Effect of active vs. passive (recorded navigation) navigation on spatial memory. No intentional encoding. Participants could freely navigate the apartment.	//	Non-immersive; input device: joystick; house apartment navigation.	Spatial layout test (spatial layout drawing of the VE); recognition task (objects); object location test.	Spatial layout recall replicated for active condition. No effect on other tasks.
[63]	30 YA (age range = 18–30) and 30 OA (age range = 58–72)	Role of active vs. passive (pre-recorded video) motor exploration in spatial memory and wayfinding. Intentional encoding. No decision-making.	Between	Non-immersive; input device: joystick; virtual replica of Bordeaux.	Wayfinding task (replication of the path; use of spatial representation, errors and stops were calculated); spatial memory task (map drawing + picture classification; route and survey representations).	Active condition worsened survey knowledge (spatial map) in both groups, led to better wayfinding scores in YA and worsened in OA. Executive functions have a crucial role during active navigation.
[64] Exp. 1	22 undergraduates (14 males)	Exploring the role of motion control vs. passive condition (VE tour) on spatial learning. Intentional encoding (learning phase before test phase). Navigation instructions were given.	Between	Non-immersive; input device: keyboard; VE of a research lab.	Spatial learning test (indicate position and direction of egocentric pictures on a lab map; object location test).	View positioning test was better for active participants, no significant difference between the conditions was observed for object location task. Active navigation contributes partially to survey representation.
[64] Exp. 2	80 undergraduates (49 males)	Exploring the role of optical flow (action with object in active condition) vs. passive navigation vs. static condition on spatial learning between active. Intentional encoding (learning phase before test phase). Navigation instructions were given.	//	Non-immersive; input device: keyboard; VE of a research lab.	Spatial learning test (indicate position and direction of egocentric pictures on a lab map; object location test).	Active participants performed better in object locations task and passive condition performed better than static condition. No difference among the conditions was observed for the first task.
[65] Exp. 1	82 university students (age range: 19–33; 43 males)	Effect of intentional vs. incidental encoding vs. active vs. passive navigation (observing the participant navigating) on spatial memory. Auditory route instructions were given (no decision-making).	Between (yoked)	Non-immersive; input devices: keyboard and mouse; virtual city.	Spatial memory test (landmark recognition task, pointing task and path-sketching, route navigation task; respectively, landmark, survey and route knowledge).	Active navigation led to better landmark and route knowledge performances. No effect on survey knowledge. No effect of encoding.
[65] Exp. 2	88 university students (age range: 18–33; 10 males)	Effect of movement (active navigation vs. passive) vs. instruction control (instructing vs. listening) vs. instruction specificity (landmark information vs. layout information) on spatial memory. Navigation instructions were written (no decision-making).	//	Non-immersive; input devices: keyboard and mouse; apartment with rooms.	Spatial memory test (landmark recognition task, tour integration task, route navigation task).	Landmark knowledge, tour integration and route knowledge benefited from self-contained condition. Effect on performance was mediate by instruction specificity and control in the latest task.
[65] Exp. 3	102 students (age range: 19–41; 21 males)	Effect of active vs. passive navigation vs. decision-making (map) vs. less decision-making (map with suggested path) vs. no-map condition on spatial memory. Participants were asked to find the shortest possible route.	//	Non-immersive; input devices: keyboard and mouse; apartment with rooms.	Spatial memory test (landmark recognition task and tour integration task; route navigation task).	Active navigation led to better landmark recognition performance. Decision-making helped participants in observed movement condition and less decision-making worsened route knowledge.
[66]	24 undergraduates (age range: 18–21; 7 males)	Effect of active free navigation (with decision-making) vs. passive on object-memory. Intentional encoding.	Between (yoked)	Non-immersive; input device: keyboard; virtual city.	Object task (locate objects) and recognition task.	No difference between the two conditions in the tasks.
[53] Exp. 1	72 undergraduates (age range: 18–27; 22 males)	Effect of psychological activity (decision-making vs. no decision-making on directions) and physical activity (motor control vs. no motor control on keyboard) vs. control group on spatial performance. No intentional encoding (explore VE).	Between	Non-immersive; input device: keyboard; virtual city.	Orientation task (direction test + map drawing).	No difference was observed between the conditions manipulated.
[53] Exp. 2	36 undergraduates (age range: 18–42)	Effect of active exploration vs. passive observation of navigation vs. control (no exploration of VE) on wayfinding. No intentional encoding (explore VE).	Between	Non-immersive; input device: keyboard; virtual arena.	Wayfinding task.	No difference was observed between the conditions manipulated.
[67]	18 YA (age range: 20–39; 9 males)	Effect of active (with decision-making) vs. passive dynamic (recorded video) vs. passive static (slide-like scenes) free exploration on spatial layout performances. Intentional encoding.	Within	Non-immersive; input device: joystick; virtual arena with cubes.	Target location test (8 trials; score, time, orientation and verbal or drawing description of the strategies used to reach a given target were calculated).	Active participants performed better than the two passive conditions in the task (scores, time, verbal and layout descriptions but no orientation task). Active motor behaviour with active perception is crucial to extract invariants in the VE.
[68]	30 YA (age range = 18–25)and 30 OA (age range = 60–81)	Active (with decision-making) vs. passive (computer-guided tour) free navigation effects on memory for everyday objects. Intentional encoding.	Between	Non-immersive; input devices: keyboard and mouse; VR-based Human Objects Memories from Everyday Scenes (HOMES).	Free recall and recognition (learning, proactive interference, semantic clustering, recognition hits, and false recognitions). VE 1A followed by free recall task + VE 2A followed by free recall task + VE B followed by recognition task.	Active navigation had a beneficial effect on recognition hits only, in both YA and OA compared with passive mode. Active mode reduced false recognitions in YA but increased these in OA. Active navigation enhanced memory in older adults when to not demanding.
[69]	44 students (mean age = 21.94, SD = 2.13; 21 males)	Active (with decision-making) vs. passive (computer-guided tour) free navigation effects on memory for everyday objects. Intentional encoding.	Between	Non-immersive; input devices: keyboard and mouse; VR-based HOMES.	Free recall and recognition (learning, proactive interference, semantic clustering, recognition hits, and false recognitions). VE 1A followed by free recall task + VE 2A followed by free recall task + VE B followed by recognition task.	Active navigation led to better recognition hits performances compared to passive condition. Active participants had less source-based false recognition compared with passive participants. Active navigation was useful to enrich visuomotor details of episodic memory traces but had no effect on semantic relational processing.
[9] Exp. 3	41 participants (age range: 18–34; 12 males)	Active vs. passive (recorded actions; no motor response) selection affects memory for object. Intentional encoding. Instructions.	Between	Non-immersive; input device: keyboard; WWW (*what*–*where*–*when*) variation built with Second Life arena.	Object name cued recall (full episodic recall: *what + where + when*; non-episodic: *where + what* or *when + what* or *what* only).	Active condition reduced distractor encoding compared to the passive viewing of the action of the avatar.
[43]	72 Psychology students (mean age = 22.23, SD = 3.94; 36 males)	Interaction condition (motor trace in memory, no decision on itinerary) vs. planning condition (no control of the vehicle; decisional level) vs. passive (recorded video). Intentional encoding.	Between	Non-immersive; input devices: steering wheel and pedals; virtual city.	Free recall of elements; visuospatial memory test (draw map + locate elements); visuospatial cued recall (locate elements on a prepared map); recognition test (elements, locations and navigation directions after seeing the elements).	Interaction enhanced memory recall, in particular spatial memory test (no effect on influence on visuospatial cued recall or recognition); however, interaction worsened elements recognition compared with passive condition; planning condition boosted visuospatial recalls. Both interaction and planning had an effect on episodic memory.
[70]	21 healthy OA (4 males), 15 aMCI (7 males) and 15 AD (2 males)	Active vs. passive (recorded video) encoding influences episodic memory. Intentional encoding. Predetermined route.	Between	Non-immersive; input devices: steering wheel and pedals; two virtual cities.	Immediate free recall (*what, details, when*, egocentric *where*, allocentric *where*, binding); recognition (elements, spatial and temporal relations between elements; remember/know paradigm); delayed free recall (same as immediate free recall).	Active exploration led in OA, aMCI, and AD groups to better recall of elements, allocentric spatial information and binding. Procedural skills and self-involvement may be crucial for episodic performances in aMCI and AD patients.
[71]	113 psychology students (mean age = 21.57, SD = 2.99) and 45 OA	Effect of active vs. passive navigation and intentional vs. incidental encoding on episodic memory. Predetermined route.	Between	Non-immersive; input devices: steering wheel and pedals; virtual city.	Free recall (*what*, verbal *where*, visuospatial *where*, *when*, *details*); recognition test (elements).	Encoding conditions affect differently episodic features in YA and OA. However, any effect due to sensorimotor implication emerged in the study.
[8]	64 YA and 64 OA (32 males)	HNC (real-life driving conditions) vs. LNC (only pedals; no enactment associated with direction) vs. IC (verbal instructions without driving; decisional level only) vs. passive (no driving no decision) effect on episodic memory performance. Intentional encoding.	Between	Non-immersive; input devices: steering wheel and pedals; virtual city.	Immediate free recall (*what* and *details*; binding: *what + where + when*; remember/know paradigm); visuospatial recall test (*what, where, when* on real map); delayed free recall test (*what*, *details*, *where*, and *when*); recognition test (elements).	Binding, regardless of age-groups, was enhanced by LNC and IC; HNC and passive conditions did not help episodic memory performance in both groups. Interestingly, Remember responses were boosted in older adults by IC condition. Active condition may be helpful when do not overload cognitive resources.
[72]	90 students (average age of 20; 45 males)	Passive VE (recorded route) vs. active VE vs. real environment (navigate the environment with instructions) and immediate vs. 48-h recall. Predetermined route.	Between	Non-immersive; input device: joystick; virtual replica of the Bordeaux area.	Immediate or 48-h recall task: real world wayfinding (replication of the real route), freehand sketch (directional changes) and photograph classification (picture in chronological order).	Transfer and sketch task are efficient after 48 h of retention and it is efficient for the two paper-pencil tasks. Active navigation led to benefits in wayfinding task, irrespective of the delay retention.
[73]	59 YA (age range: 19–29; 19 males)	Effect of active vs. passive free exploration on object recognition in VEs.	Between	Immersive; input device: keyboard; virtual rooms with objects.	Recognition task (objects).	Active navigation led to higher hit and lower miss responses than the passive condition. Active navigation has an important role in landmark recognition.
[74] Exp. 1	32 YA (age range: 18–34; 16 males)	Active vs. passive free navigation with four trials with different virtual maze.	Between	Non-immersive with static navigation; input devices: keyboard and mouse; virtual maze.	“Active” test: number of moves and time.	Navigational knowledge is represented regardless the kind of exploration condition.
[58]	20 male students (age range: 20–26)	Active (self-governed) vs. passive (avatar-guided) free exploration. Four exploring sessions. Intentional encoding.	Between	Immersive (HMD); input device: joystick; virtual school.	Wayfinding task (short route to starting point); pointing task (orienting to the starting point); sketch-map (local accuracy or survey-type organization).	Self-governed explorers were better in completing the wayfinding task. Sketch-map accuracy was similar in both groups, whereas self-governed group had better survey-type organization. No differences were shown in pointing task. Self-governed participants organize their knowledge in survey mode.
[75]	34 YA (age range: 18–38; 7 males)	Active vs. passive (passenger condition) condition. Participants were before divided in driver and non-drivers. Intentional encoding and decision-making.	Between	Non-immersive: input devices: steering wheel and pedals; virtual city.	Survey knowledge: pointing error scores (street-level view) and map placement error scores (bird’s eye view); route knowledge: route scores (shortest route).	Driver had better route scores during active navigation compared with drivers in passive and non-driver in active conditions. Drivers showed better map scores (no condition effect). Active navigators do not learn more spatial layout knowledge and dual task effect may affect scores in non-drivers.
[59]	54 students (9 males)	Active vs. passive (passenger condition). Three exposures (3, 10, or 15 times). No intentional encoding. Predetermined path.	Between (yoked)	Non-immersive: input devices: steering wheel and pedals; virtual city.	Survey knowledge (map sketch drawing and map rates’ score), route knowledge (travel directions) and landmark knowledge (landmarks recall).	Passengers recalled more landmarks across exposure conditions. Survey errors reduced between 5 and 15 times in both conditions. Exposure led to better map reliability especially for the passive condition. Attentional resources could have led to worst performance in active drivers.
[76] Exp. 3	41 undergraduates (age range: 18–24; 20 males)	VE active vs. VE passive (watch experimenter navigation) vs. VE + line (active with path to follow; no free exploration) vs. control (no VE training; real -world wayfinding).	Between	Non-immersive. Input device: keyboard; virtual replica of an office.	Real world transfer task (balloons wayfinding times and errors from virtual to real places); training task (wayfinding time and errors in VE conditions).	Times for active condition were lower compared with control condition and active and VE + line led to fewer errors than control condition. Virtual real transfer occurs thanks to virtual interaction.
[60]	64 students (average age of 20; 32 males)	Ground vs. areal point of view and active vs. passive navigation. Predetermined route	Between	Non-immersive; input device: joystick; virtual replica of the Bordeaux area.	Real world wayfinding task (replication of the real route; error scores), sketch-drawing task (directional changes; errors and omissions scores) and scene-sorting task (errors).	Active navigation boosted sketch-mapping task and worsened wayfinding and picture-sorting scores. Grounded-level condition improved performance in wayfinding and picture-sorting tasks, whereas aerial-level in sketch-mapping task. Active navigation and grounded-level interaction had a positive effect in the wayfinding and picture-sorting tasks, whereas passive and aerial-level condition improved sketch-mapping scores. Egocentric information and motor information create a correct perception-action coupling.
[77]	64 students (average age of 20; 32 males)	Detailed vs. undetailed visual fidelity and active vs. passive navigation. Predetermined route.	Between	Non-immersive; input device: joystick; virtual replica of the Bordeaux area.	Real-world wayfinding task (replication of the real route; errors and hesitations scores), sketch-mapping task (directional changes; errors and omissions scores), and scene-sorting task (errors).	Results highlighted better performance for each spatial task in both active and detailed condition. Interaction effect (active and detailed) led to better scores for sketch task and active condition combined with undetailed VE worsened scene-sorting task. Perceptual-motor information is crucial in spatial knowledge. Visual fidelity has positive effect for allocentric representation but not for route knowledge.
[78] Exp. 1 & 2	28 students (age range: 18–23; 9 males)	Active exploration (experiment 1) vs. passive exploration (experiment 2; video of active exploration). Predetermined route of familiar environment.	Between	Non-immersive; input device: keyboard; university building.	Orientation test trials of external cues from four virtual rooms (internal visited and unvisited, external visited and unvisited).	There is no difference in the two conditions.
[78] Exp. 3	54 visitors (mean age = 17.55, SD = 1.14; 19 males)	Active exploration vs. passive exploration. Predetermined route of unfamiliar environment.	Between	Non-immersive; input device: keyboard; university building.	Orientation test trials of external cues from four virtual rooms (internal visited and unvisited, external visited and unvisited).	No effect of unfamiliarity for active participants. Passive participants had greater error for the internal unvisited room. Active exploration enhances survey knowledge for unfamiliar environments.
[79]	60 adults (mean age = 25.2, SD = 4.5; males 49)	Active exploration vs. passive exploration (video of passive exploration) and immersive vs. computer screen. Predetermined rout of Gowanus canal. Intentional encoding. An allocentric map of the canal was provided in all conditions.	Within	Immersive (Emotiv EPOC headset) and non-immersive; input device: mouse and headset gyroscope; Gowanus Canal.	Elements recognition task	No difference between the two navigation conditions. However, active navigation with the mouse has higher level of engagement.
[80] Exp. 1 and 2		3D active exploration (Exp. 1) vs. 2D passive snapshots presentation (Exp. 2). Free exploration and intentional encoding.	Between	Immersive (nVisor SX111) and non-immersive; input device: Wiimote; virtual apartment.	Search trials of geometric and contextual objects.	Search task improved in both conditions but in the immersive condition initial fixations and time spent in the incorrect rooms and better selection of the correct room indicate higher use of memory.
[81]	28 YA (mean age = 25.6, SD = 5.4; 17 males)	Active navigation vs. passive navigation (video). Immediate (intentional encoding) and 24 h delayed (naïve) recall. Participants could freely navigate the environment.	Between	Non-immersive; input device: keyboard and mouse; virtual city.	Immediate and delayed free recall of semantically linked images of 3D objects placed in the town.	No effect of navigation types on spatial memory.
[57]	14 YA (mean age = 22, SD = 2.08; 7 males)	Full condition (full control over the navigation) vs. medium condition (participants move but do not control pre-recorded navigation) vs. low condition (watch pre-recorded navigation).	Within	Immersive (Oculus Rift DK2); input device: Kinect for legs and arms movement detection; virtual city.	Immediate free recall (*what*, egocentric *where*, *details*, *when*, *binding*) and item recognition (source memory, remember/know/guess paradigm), egocentric, allocentric and temporal recognition.	Any significance was found among the conditions. However, the full and medium (virtual embodiment) conditions were more immersive than the passive one.
[82]	16 students (females = 16)	Active navigation vs. passive (watching the navigation of the active participant). Free exploration.	Between (yoked)	Non-immersive (46-inch touchscreen monitor); input device: joystick; virtual rooms	Immediate memory recognition for objects manipulated in each room).	Both passive and active navigation had a significant negative effect on memory of object, with active navigation having a greater effect compared to passive.
[61]	22 YA (mean age = 19.71, SD = 2.19; females = 11) and 22 OA (mean age = 74.55, SD = 7.82; females = 10)	Active navigation vs. passive. Free exploration.	Within (yoked)	Immersive (cardboard) mobile application (input device: button headset and head movements); VE (city, park, mall)	Encoding-Retrieval route overlap accuracy	Active encoding leads to better spatial memory in OA; accuracy is predicted by age, active exploration and visuospatial abilities.
